# Productive activities, mental health and quality of life in disability: exploring the role enhancement and the role strain hypotheses

**DOI:** 10.1186/s40359-018-0276-6

**Published:** 2019-01-08

**Authors:** Christine Fekete, Johannes Siegrist, Marcel W. M. Post, Martin W. G. Brinkhof, for the SwiSCI Study Group, Xavier Jordan, Bertrand Léger, Michael Baumberger, Hans Peter Gmünder, Armin Curt, Martin Schubert, Margret Hund-Georgiadis, Kerstin Hug, Thomas Troger, Daniel Joggi, Nadja Münzel, Hardy Landolt, Mirjam Brach, Gerold Stucki, Christine Thyrian, Christine Fekete

**Affiliations:** 1 Swiss Paraplegic Research, Guido A. Zäch Institute, 6207 Nottwil, Switzerland; 2 Department of Health Sciences and Health Policy, University of Lucerne, Frohburgstrasse 3, 6002 Lucerne, Switzerland; 3 Senior Professorship ‘Work Stress Research’, Faculty of Medicine, University of Duesseldorf, Life-Science-Center, Merowingerplatz 1a, 40225 Duesseldorf, Germany; 4 Department of Rehabilitation Medicine, University of Groningen, University Medical Center Groningen, Hanzeplein 1, 9713 Groningen, the Netherlands; 5 Center of Excellence for Rehabilitation Medicine, Brain Center Rudolf Magnus, University Medical Center Utrecht, Utrecht University and De Hoogstraat Rehabilitation, Universiteitsweg 100, 3584 CG Utrecht, the Netherlands

**Keywords:** Productive activities, Mental health, Quality of life, Role enhancement, Role strain, Disability, Spinal cord injuries

## Abstract

**Background:**

Engagement in productive activities is an important determinant of mental health and quality of life (QoL). Persons with physical disabilities are often confronted with constraints to engage in productive activities and it remains largely unknown whether persons who nevertheless manage to be productive experience beneficial effects for mental health and QoL. This is the first study to analyse different productive activities (paid work, volunteering, education, housework) and its gender-specific associations with mental health and QoL in the disability setting, testing two contrasting hypotheses of Role Theory, the role strain and the role enhancement hypotheses.

**Methods:**

We used data from a representative sample of 1157 men and women of employable age who sustained a severe physical disability (spinal cord injury). *Load* of engagement in paid work, volunteering, education, and housework was classified into three groups (none; moderate; high). To assess the total productivity load, a score over the four items was calculated. *Diversity* of engagement was assessed with variables on the number and combination of activities. Tobit regressions were applied to evaluate associations of load and diversity of engagement in productive activities with mental health (Mental Health Inventory, SF-36) and QoL (WHOQoL-BREF items).

**Results:**

We found that the total productivity load and the load of paid work were positively related to mental health and QoL in men. Individuals with moderate engagement in volunteering reported better mental health (both genders) and QoL (in women) than those with higher or no engagement. Our results support the role enhancement hypothesis, as mental health (in men) and QoL (both genders) increased with the number of performed activities. In men who had paid work, mental health and QoL increased consistently with each additional unpaid activity. In contrast, engagement in paid work played a minor role for mental health and QoL in women.

**Conclusion:**

This study in the disability setting provided clear support for the role enhancement hypothesis. Future research on the mechanisms behind the observed associations is warranted to develop interventions and policies that strengthen resources important for engagement in productive activities as well as for mental health and QoL in persons with physical disabilities.

## Background

Engagement in productive activities is an important determinant of mental health and quality of life (QoL) [1–4]. Evidence suggests that engagement in activities such as paid work, housework, volunteering or education provides opportunities for the fulfilment of basic human needs [5] that are essential for the maintainance of mental health and QoL [6]. Basic needs include feelings of belonging and social affiliation [7], development and maintenance of skills and competences [6, 8], and recognition and appreciation from significant others [9, 10]. Conversely, such basic needs may remain unfulfilled if people are deprived from engagement in productive activities. Consequences from unmet needs may be social exclusion [11], lack of autonomy [12] and social reward deficiency [13], which negatively affect mental health and QoL.

Role Theory provides two contrasting postulates for the link between engagement in a diversity of productive activities and health, namely the role enhancement and the role strain hypotheses. The *role enhancement* hypothesis states that the simultaneous availability of different roles and distinct role combinations through a diversity of role engagements is likely to strengthen personal need fulfilment [14]. Engagement in diverse roles leads to a broad range of interactions with others that may offer sources for socio-emotional support [15] and different types of rewards [16], which in turn positively affect mental health and QoL. In contrast, the *role strain* hypothesis states that engagement in diverse roles may result in role overload and burden as different obligations hinder successful role performance [17]. Unfulfilled role obligations may elicit stress reactions that negatively affect mental health and QoL. The two hypotheses of productive activities and their associations with mental health and QoL have been studied in general populations only [1–4, 14, 18, 19]. Results of these studies were inconclusive, which may be related to variations in constraints on productive activities across study populations, for instance variation in functional capacity of study participants or differences in labour market accessibility.

**Fig. 1:**
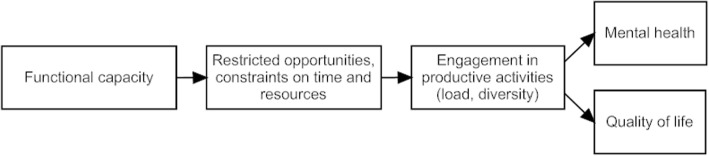
Productive activities, mental health and quality of life in the disability setting

In this study, we tested the two contrasting hypotheses of Role Theory in the context of physical disability. Populations with varying degrees of functional capacity typically show heterogeneous levels of engagement in productive activities. Depending on the functional capacity, persons with physical disabilities are confronted with varying constraints on time, resources and opportunities to engage in productive activities, most obviously with regard to paid work [20]. More specifically, persons with physical disabilities are often forced to reduce the amount of productive activities and are likely to spend higher energy efforts to meet role demands. Based on the within-sample variation in functional capacity and resulting constraints on engagement, we expect that the associations between load and diversity of productive activities and mental health or QoL are more pronounced in populations with physical disabilities than in general populations (Fig. 1). Whether these barriers negatively impact on mental health and QoL or whether individuals who manage to overcome constraints and are able to engage in productive activities profit from beneficial effects remains to be tested [21, 22].

The overall objective of this study is therefore to investigate the associations of engagement in productive activities with mental health and QoL in a population-based sample of men and women with a physical disability in the employable age. Spinal cord injury (SCI) may offer an informative case to assess these associations in-depth, as this condition is characterised by varying degrees of functional capacity, depending on the injury severity. An SCI is a damage to the spinal cord of traumatic or non-traumatic origin that causes a total or partial loss of sensation and movement below the lesion level. Given their pronounced functional limitations, persons with SCI often face environmental barriers to engagement in productive activities, such as inaccessible infrastructure or negative attitudes [23–25]. While figures on engagement in unpaid productive activities in SCI are widely lacking, participation in paid work in persons with SCI is well described. The average global employment rate is estimated at 37% [26], with considerable variations between countries, ranging from 11.5% to 74% [27]. Findings for SCI populations may also apply to other types of physical disabilities, specifically those characterize by mobility limitations and dependency [28].

The specific aims of this study are 1) to investigate the association of load of engagement in four different types of productive activities with mental health and QoL and 2) to explore two contrasting hypotheses, the role enhancement and the role strain hypothesis, by investigating the association of engagement in a diversity of productive activities with mental health and QoL in persons with a physical disability. The positive association of diversity in productive activities and mental health and QoL would support the role enhancement hypothesis, while a negative association would lend support to the role strain hypothesis. Given traditional gender roles and gender-specific occupational experiences and qualifications [29–31], engagement in productive activities varies substantially by gender [24, 32]. For example, there are substantial gender differences in the prevalence and subjective importance of paid and unpaid productive engagement, supporting the importance to perform separate analysis for men and women.

## Methods

### Design

We analysed cross-sectional data from the population-based community survey of the Swiss Spinal Cord Injury Cohort Study (SwiSCI) [33]. The SwiSCI survey is the largest European survey on persons with SCI to date, and was conducted between late 2011 and early 2013. Data were collected by paper-pencil or online questionnaire, and in special cases, telephone interviews [34, 35]. After a written invitation, up to two written reminders and a telephone call were implemented to maximize response rates. Further details on study design, recruitment procedures and reminder management are given elsewhere [34, 35].

This study has been approved by the Medical Ethical Committee of the Canton Lucerne, Switzerland (document 11,042), and subsequently by the Ethics Committees of all other involved Swiss cantons, namely Basel (document 306/11) and Valais (document 042/11). In addition, the study protocol has been approved by the Steering Committee of the SwiSCI study and all participants have signed a written consent form.

### Sampling frame and study participants

The SwiSCI community survey included Swiss residents with a traumatic or non-traumatic SCI aged over 16 years. Exclusion criteria were congenital conditions leading to SCI, new SCI in the context of palliative care, neurodegenerative disorders, and Guillain-Barré syndrome. Given the lack of a central registry covering all persons with SCI in Switzerland, the SwiSCI population was recruited through the national association for persons with SCI (Swiss Paraplegic Association), three specialized SCI-rehabilitation centers, and a SCI-specific home care institution [33]. Of 3144 eligible persons, 1549 completed the first two questionnaires relevant for this study (cumulative response rate 49.3%). We found minimal response bias in relation to key characteristics such as gender, age and lesion severity, indicating that the SwiSCI sample good representation of the sampling frame [34]. The sample of the present study was restricted to 1198 persons in employable age. The lower age limit of 16 years was defined by the inclusion criteria of the study and the fact that many adolescents start an apprenticeship at the age of 16, which is to be considered as first paid employment. The upper age limit was defined by the legal age of employment in Switzerland (< 65 for men, < 64 for women). We only included men and women in employable age for whom information on mental health and QoL was available (*n* = 1157). Further details on recruitment outcomes, participation rates, and non-response bias in the SwiSCI community survey 2012 can be found elsewhere [34, 35].

### Measures

*The load of engagement in productive activities* was assessed with the Utrecht Scale of Evaluation in Rehabilitation-Participation (USER-P), which consists of three subscales on participation frequency, restrictions, and satisfaction [36]. We used the USER-P frequency subscale that includes four items on the load of paid work, volunteering (activities in clubs, community institutions or other volunteering), education, and housework. The load of engagement was assessed with a six-point scale on ranges of hours per week (0; 1–8; 9–16; 17–24; 25–35; > 35). To calculate the total productivity load, the categories on engagement load were coded as follows: 0 = 0 h; 1 = 1–8 h; 2 = 9–16 h; 3 = 17–24 h; 4 = 25–35 h; and 5 = > 35 h. Based on this coding and in accordance with recommendations from the USER-P developers [36], we calculated a score ranging from 0 to 100 to assess the total productivity load (sum score of all productivity variables multiplied by 5). We categorized the total productivity load into distribution-based quartiles for analysis.

The response scales of the single items on load of engagement in productive activities were categorized into none, moderate, and high based on the response distributions. For paid work, moderate load was defined as 1–16 h/week, high load as > 16 h/week. For volunteering, education and housework, moderate load was defined as 1–8 h/week, and high load as > 8 h/week.

*Engagement in a diversity of productive activities* was evaluated by variables on the number and the combination of different activities. Number of activities was assessed by simply adding the number of productive activities in which a person was engaged in (0 ‘none of the activities’ to 4 ‘all of the activities’). To reduce the amount of possible combinations of activities, it is conceptually meaningful to distinguish between paid and unpaid activities (housework, volunteering, education). Five mutually exclusive categories reflecting different combinations of paid and unpaid activities were defined: No or one productive activity; 2–3 unpaid activities; paid work and 1 unpaid activity; paid work and 2 unpaid activities; paid work and 3 unpaid activities. The category ‘paid work only’ was rare (*n* = 28) and thus not analyzed separately.

*Mental health* represents a multidimensional construct of disease orientated symptoms [37], whereas *QoL* is used as overall concept to describe the subjective appraisal of a persons’ health, mood and satisfaction with life [38]. Mental health was assessed with the five-item Mental Health Inventory of the 36-item Short Form Health Survey (MHI-5 SF-36, version 1) [39]. The MHI-5 assesses the frequency of mood states in the past four weeks on a six-point scale. Its empirical validity and reliability is supported for SCI populations [40]. A sum score ranging from 0 to 100 was calculated according to established algorithms [41], with higher scores indicating better mental health. *Quality of life* was assessed with five WHOQoL BREF items [42]. The items assess people’s perception of their overall QoL and satisfaction with health, social relationships, activities of daily living, and living conditions. Satisfactory psychometric properties have been demonstrated for SCI populations [43]. A sum score ranging from 0 to 20 was built over the five items, with higher scores indicating better QoL.

#### Potential confounders

Given their established association with the ability to engage in productive activities as well as with mental health and QoL, sociodemographic (age, education, receipt of disability pension) and lesion characteristics (years since injury, level and completeness of lesion, aetiology) were included as potential confounders [24, 44, 45]. To obtain unbiased estimates of the associations, we additionally control for functional capacity [45]. Besides level and completeness of lesion, we therefore included Rasch-based scores of the Spinal Cord Injury Independence Measure for Self-Report (SCIM-SR) [46, 47] as indicator for functional independence. Acute health conditions were measured with a 14-item scale on the frequency and severity of common SCI-related health conditions (e.g., spasticity, urinary tract infections, pain, sleep problems). These health conditions were not included as confounders into analysis as we cannot test whether their occurrence leads to reductions in productive activities or sickness absence or whether people have this condition chronically, with no impact on their current productive engagement.

### Statistical analysis

Analyses were conducted using STATA version 14.0 for Windows (College Station, TX, USA). All analyses were stratified for men and women. Where applicable, missing values in the four items on productivities activities were complemented by available information on the current employment situation (paid work, yes/no; workload in percent of full time equivalent; in education, yes/no; housewife, houseman, yes/no). Persons with remaining missing values in the four productivity items were excluded from multivariable analyses (*n* = 22 men, *n* = 12 women). Missing values in potential confounders were accounted for using multiple imputation (MI) by chained equations (MICE), imputing categorical, ordinal and linear variables in one model [48, 49]. For each model, 20 imputed datasets were created. Multivariable models were weighted for unit-nonresponse, using inverse probability weights for the SwiSCI population in the employable age [34].

Crude distributions of engagement in productive activities, potential confounders, mental health, and QoL are presented. Further, cross-tabulations were performed to investigate unadjusted associations of load and diversity of engagement in productive activities with mental health and QoL. We report mean and standard deviations (SD) of the mental health and QoL scores across the categories and provide *p*-values of Kruskal-Wallis tests and Cuzicks’ tests for trend [50] to evaluate the difference between categories and the ordering of estimates between groups.

Tobit regression was applied to evaluate the association between the ‘predictors’ (total productivity, load of paid work, volunteering, education, housework; engagement in a diversity of productive activities: number and combination of activities) and the ‘outcomes‘(mental health; QoL). Tobit models were chosen to account for the right censoring in the continuous scores on mental health and QoL [51]. Adjusted models were controlled for sociodemographics, lesion characteristics and functional independence. Additionally, the models on load of paid work, volunteering, education and housework were mutually controlled for the load of other activities. For example, the model using load of paid work as main predictor was additionally adjusted for the load of volunteering, education and housework. As sensitivity analysis, models for QoL were controlled for mental health, as mental health may also affect productivity. Although the adjustment for mental health does not solve the issue of directionality of relationships, it enables to explore whether productivity is related to QoL, independently of mental health. All variables on productive activities were entered as categorical variables as described in the ‘Measures’ section using the group with the lowest engagement or no engagement as reference group.

To explore gender differences in associations, interactions between gender and productive activities were tested. Significant interaction terms indicate differences in the association between productive activities and mental health or QoL in males and females (differences in slopes) and provide evidence for a moderating effect of gender in the studied association.

In respective Tables and Figures, *β* coefficients, 95% confidence intervals (CI), and *p-*values from equal fraction-missing-information (FMI) tests are provided. The FMI is an indicator for variance attributable to missing data. In FMI tests, it is assumed that the between-imputation variance is proportional to the within-imputation variance and subsets of variables are tested for significance by jointly testing whether coefficients equal zero [52]. *P*-values of FMI-tests can be interpreted similarly to other *p-*values, i.e. values below 0.05 are considered to indicate a significant association between a predictor and an outcome.

## Results

Basic characteristics of the study participants are given in Table 1. The majority of the sample were men (72%), with a mean age of about 46 years in both genders. Paraplegia was the most prevalent diagnosis and the majority of injuries were caused by a traumatic event. On average, people had lived 17 years with SCI. Gender differences were observed for the load of engagement in productive activities with men being more often involved in paid work and education and less often in housework than women. The total productivity was similar for both genders, however, the mean number of productive activities was somewhat higher in men. Concerning the combination of activities, women were more often engaged in unpaid activities, whereas the combination of paid work and two or more unpaid activities was more prevalent in men. Men showed higher scores of mental health than women, while gender differences in QoL were small and insignificant.

**Table 1:** Basic characteristics of the SwiSCI baseline population in employable age

	m: men, women	Total	Men	Women	*p* gender differences^b^
		(*n* = 1157)	(*n* = 840)	(*n* = 317)	
Sociodemographic and lesion characteristics					
Age, mean (SD)	0, 0	46.5 (11.2)	46.7 (11.2)	45.8 (11.2)	*0.225*
Education in years, mean (SD)	15, 2	13.9 (3.3)	13.9 (3.2)	13.8 (3.4)	*0.069*
Having a partner, n (%)	28, 6	747 (66.5)	552 (68.1)	195 (62.7)	*0.093*
Receiving disability pension, n (%)	0, 0	648 (56.0)	446 (53.1)	202 (63.7)	*0.001*
Lesion severity, n (%)	7, 2				*0.001*
Paraplegia incomplete		400 (34.8)	268 (32.2)	132 (41.9)	
Paraplegia complete		392 (34.2)	293 (35.2)	99 (31.4)	
Tetraplegia incomplete		223 (19.4)	160 (19.2)	63 (20.0)	
Tetraplegia complete		133 (11.6)	112 (13.5)	21 (6.7)	
Time since injury in years, mean (SD)	17, 8	16.9 (11.7)	17.0 (11.8)	16.6 (11.6)	*0.674*
Traumatic aetiology, n (%)	10, 3	958 (83.7)	728 (87.7)	230 (73.3)	*< 0.001*
Non-traumatic aetiology		186 (16.3)	102 (12.3)	84 (26.8)	
Functional independence, 0-100, mean (SD)	205, 86	66.6 (22.0)	67.1 (22.2)	67.7 (19.8)	*0.912*
Load of productive activities					
Total productivity load, 0–100, mean (SD)	22, 12	22.9 (12.7)	23.2 (12.9)	22.2 (11.9)	*0.188*
Lowest quartile, n (%)		264 (23.6)	187 (23.0)	77 (25.4)	*0.266*
2nd quartile, n (%)		259 (23.2)	179 (22.0)	80 (26.4)	
3rd quartile, n (%)		328 (29.4)	247 (30.3)	81 (26.7)	
Highest quartile, n (%)		266 (23.8)	201 (24.7)	65 (21.5)	
Paid work (h/week), n (%)	1, 1				*< 0.001*
None		446 (39.1)	290 (34.9)	156 (50.2)	
1–16		195 (17.1)	131 (15.6)	69 (21.8)	
> 16		501 (43.9)	418 (49.8)	91 (28.8)	
Volunteering (h/week), n (%)	0, 1				*0.066*
None		572 (49.5)	398 (47.4)	174 (55.1)	
1–8		461 (39.9)	349 (41.6)	112 (35.4)	
> 8		123 (10.6)	93 (11.1)	30 (9.5)	
Education (h/week), n (%)	1, 1				*0.009*
None		852 (73.8)	599 (71.4)	253 (80.1)	
1–8		240 (20.8)	188 (22.4)	52 (16.5)	
> 8		63 (5.5)	52 (6.2)	11 (3.5)	
Housework (h/week), n (%)	20, 11				*< 0.001*
None		144 (12.8)	127 (15.5)	17 (5.6)	
1–8		556 (49.4)	444 (54.2)	112 (36.6)	
> 8		426 (37.8)	249 (30.4)	177 (57.8)	
Diversity of productive activities					
Number of productive activities, mean (SD)	22, 12	2.3 (1.1)	2.3 (1.1)	2.1 (0.9)	*0.001*
None, n (%)		55 (4.9)	48 (5.9)	7 (2.3)	*< 0.001*
One, n (%)		217 (19.3)	138 (16.9)	79 (25.9)	
Two, n (%)		365 (32.5)	253 (30.9)	112 (37.7)	
Three, n (%)		349 (31.1)	259 (31.7)	90 (29.5)	
Four, n (%)		137 (12.2)	120 (14.7)	17 (5.6)	
Combination of productive activities, n (%)	22, 12				*< 0.001*
None or one activity		272 (24.2)	186 (22.7)	86 (28.2)	
Two or three unpaid activities^a^		185 (16.5)	116 (14.2)	69 (22.6)	
Paid work + one unpaid activity^a^		224 (20.0)	165 (20.2)	59 (19.3)	
Paid work + two unpaid activities^a^		305 (27.2)	231 (28.2)	74 (24.3)	
Paid work + three unpaid activities^a^		137 (12.2)	120 (14.7)	17 (5.6)	
Mental health and quality of life					
Mental health, 0–100, mean (SD)	0, 0	71.6 (17.8)	73.1 (17.3)	67.5 (18.4)	*< 0.001*
Quality of life, 0–20, mean (SD)	0, 0	13.6 (3.6)	13.8 (3.6)	13.4 (3.7)	*0.152*

^a^Unpaid activities are: housework, volunteering, and education. Abbreviations: *m* Missing values. % excluding missing values

^b^*p*-values from chi-square test for categorical variables (applies for all categories) and from Mann-Whitney U test for the comparison of means

### Study aim 1: Load of engagement in productive activities, mental health and QoL

In men, the total productivity load and the load of paid work were positively linked to mental health and QoL in unadjusted analysis. Men with moderate engagement (1–8 h/week) in volunteering and education reported better mental health and QoL than those with higher or no engagement. In women, the total productivity load and the load of engagement in paid work and volunteering were consistently related to QoL, but not to mental health (Table 2).

**Table 2:** Unadjusted associations of load of engagement in productive activities, mental health and quality of life for men and women, mean (SD)

	Men		Women	
	Mental health	Quality of life	Mental health	Quality of life
	Mean (SD)	Mean (SD)	Mean (SD)	Mean (SD)
Total productivity load				
Lowest quartile	67.2 (20.0)	11.9 (3.6)	61.7 (20.8)	11.5 (4.2)
2nd quartile	73.5 (16.2)	13.4 (3.2)	70.7 (16.2)	13.7 (3.2)
3rd quartile	74.7 (15.6)	14.4 (3.2)	68.1 (16.4)	13.9 (3.6)
Highest quartile	76.6 (15.8)	15.0 (3.5)	68.6 (18.7)	14.6 (3.4)
*p*	*< 0.001; < 0.001*	*< 0.001; < 0.001*	*0.041; 0.102*	*< 0.001; < 0.001*
Paid work (h/week)				
None	67.8 (19.3)	12.2 (3.7)	66.1 (18.8)	12.8 (3.9)
1–16	73.3 (15.7)	13.7 (3.1)	65.8 (19.6)	13.7 (3.7)
> 16	76.7 (15.3)	14.9 (3.3)	71.3 (16.2)	14.3 (3.4)
*p*	*< 0.001; < 0.001*	*< 0.001; < 0.001*	*0.093; 0.056*	*0.004; 0.001*
Volunteering (h/week)				
None	70.5 (18.9)	13.2 (3.8)	65.2 (19.0)	12.8 (3.8)
1–8	76.4 (14.3)	14.3 (3.2)	70.3 (17.0)	14.1 (3.4)
> 8	71.7 (18.7)	13.9 (3.6)	69.6 (17.6)	14.1 (4.2)
*p*	*< 0.001; 0.015*	*< 0.001; 0.003*	*0.081; 0.059*	*0.009; 0.003*
Education (h/week)				
None	72.2 (17.8)	13.6 (3.6)	67.3 (18.4)	13.2 (3.8)
1–8	76.3 (15.2)	14.4 (3.5)	67.8 (19.0)	14.1 (3.3)
> 8	72.2 (18.1)	13.9 (3.9)	72.0 (17.2)	15.5 (3.2)
*p*	*0.019; 0.081*	*0.010; 0.012*	*0.818; 0.562*	*0.091; 0.031*
Housework (h/week)				
None	70.3 (20.7)	12.4 (3.6)	64.0 (20.1)	11.7 (4.5)
1–8	74.4 (16.4)	14.2 (3.4)	68.7 (18.8)	13.3 (4.0)
> 8	72.3 (16.4)	13.7 (3.8)	66.8 (17.8)	13.6 (3.4)
*p*	*0.116; 0.796*	*< 0.001; 0.015*	*0.395; 0.721*	*0.226; 0.148*

*Note: p-*values for the comparison of means across categorical variables from Kruskal-Wall tests and Cuzicks’ tests for trend across ordered groups. Only full cases in this table

Adjusted analyses showed a positive association of total productivity load and load of paid work with mental health and QoL in men, while associations were less consistent in women (Table 3). Moderate engagement in volunteering (1–8 h/week) was related to better mental health (both genders) and QoL (in women) in comparison to higher (> 8 h/week) or no engagement. With the exception of a positive association between education and QoL in women, the load of engagement in education and housework were neither related to mental health nor to QoL. Sensitivity analyses showed that the load of engagement in productive activities was related to QoL even after adjustment for mental health (Table S1, Electronic Supplementary Material). Gender did not moderate the association (test for interactions, all *p*-values > 0.29). In case of paid work, there was weak support for a stronger association in men than in women (*p* = 0.09 for mental health; *p* = 0.06 for QoL).

**Table 3:** Load of engagement in productive activities, mental health, and quality of life: adjusted coefficients and its 95% confidence intervals (CI) from tobit regressions for men (*n* = 818) and women (*n* = 305)

	Men		Women	
	Metal health	Quality of life	Metal health	Quality of life
	Coeff (95% CI)	Coeff (95% CI)	Coeff (95% CI)	Coeff (95% CI)
Total productivity load				
Lowest quartile	Reference	Reference	Reference	Reference
2nd quartile	5.56 (1.67–9.46)	1.15 (0.42–1.87)	9.32 (3.21–15.43)	2.15 (0.96–3.33)
3rd quartile	5.45 (1.69–9.20)	1.69 (0.98–2.41)	6.10 (−0.48–12.68)	1.94 (0.59–3.28)
Highest quartile	6.99 (2.38–11.60)	2.25 (1.34–3.17)	5.45 (−2.18–13.08)	2.34 (0.96–3.72)
*p*	*0.010*	*< 0.001*	*0.029*	*0.002*
Paid work (h/week)				
None	Reference	Reference	Reference	Reference
1–16	4.32 (0.51–8.13)	1.19 (0.50–1.89)	−2.0 (−8.27–3.47)	0.20 (−0.87–1.28)
> 16	6.06 (2.68–9.44)	1.85 (1.17–2.53)	3.91 (−2.40–10.22)	0.70 (− 0.57–1.96)
*p*	*0.002*	*< 0.001*	*0.164*	*0.556*
Volunteering (h/week)				
None	Reference	Reference	Reference	Reference
1–8	3.85 (1.25–6.46)	0.49 (−0.03–1.01)	6.09 (1.64–10.55)	1.50 (0.59–2.40)
> 8	0.05 (−4.74–4.85)	0.26 (− 0.59–1.11)	5.48 (− 1.65–12.61)	1.48 (− 0.17–3.12)
*p*	*0.009*	*0.177*	*0.021*	*0.004*
Education (h/week)				
None	Reference	Reference	Reference	Reference
1–8	1.09 (−1.83–4.01)	0.18 (−0.41–0.76)	0.56 (−5.12–6.24)	1.28 (0.10–2.45)
> 8	−0.71 (−7.10–5.67)	0.43 (− 0.88–1.75)	4.42 (−4.80–13.63)	2.11 (0.05–4.18)
*p*	*0.732*	*0.714*	*0.641*	*0.024*
Housework (h/week)				
None	Reference	Reference	Reference	Reference
1–8	1.91 (−2.91–6.73)	0.44 (−0.33–1.21)	2.30 (−8.21–12.82)	0.50 (− 2.10–3.10)
> 8	0.78 (−4.40–5.96)	0.33 (− 0.52–1.19)	0.36 (− 9.84–10.57)	0.93 (− 1.61–3.47)
*p*	*0.579*	*0.536*	*0.567*	*0.576*

*p*-values from equal Fraction Missing Information (FMI) test. *Note:* Confounders imputed by multiple imputation, results weighted for unit non-response. Models are adjusted for age, receipt of disability pension, partnership, years of education, lesion severity, time since injury, aetiology, and functional independence. Models on paid work, volunteering, education, and housework are mutually controlled for each other

### Study aim 2: Diversity of productive activities, mental health and QoL

Unadjusted analyses indicated a positive association of number of productive activities with mental health and QoL in both genders (Table 4). In men, those who combined paid work and unpaid activities reported better mental health and QoL than those only performing unpaid activities or no activities. In women, the combination of paid and unpaid work seems less beneficial for mental health and QoL as scores were higher in women with an accumulation of unpaid activities. However, women engaged in all four productive activities indicated highest mental health and QoL.

**Table 4:** Unadjusted associations of diversity of productive activities, mental health and quality of life for men and women, mean (SD)

	Men		Women	
	Mental health	Quality of life	Mental health	Quality of life
	Mean (SD)	Mean (SD)	Mean (SD)	Mean (SD)
Not engaged in diverse productive activities (none or one activity)	67.1 (19.8)	11.8 (3.7)	63.3 (19.4)	11.7 (4.0)
Number of productive activities				
Two	72.7 (17.3)	13.9 (3.4)	68.3 (18.2)	13.9 (3.2)
Three	75.8 (15.0)	14.5 (3.3)	69.2 (16.8)	14.1 (3.7)
Four	77.6 (14.3)	15.0 (3.4)	70.6 (19.6)	14.8 (3.2)
*p*	*< 0.001*	*< 0.001*	*0.035*	*< 0.001*
Combination of productive activities				
Two to three unpaid activities	71.7 (17.1)	13.1 (3.5)	69.5 (17.7)	14.1 (3.3)
Paid work + one unpaid activity	73.2 (17.1)	14.3 (3.3)	68.5 (18.8)	14.0 (3.3)
Paid work + two unpaid activities	76.4 (15.0)	14.7 (3.2)	68.1 (16.5)	13.9 (3.7)
Paid work + three unpaid activities	77.6 (14.3)	15.0 (3.4)	70.6 (19.6)	14.8 (3.2)
*p*	*< 0.001*	*< 0.001*	*0.117*	*< 0.001*

*p-*values for the comparison of means across categorical variables from Kruskal-Wall test. Note: Unpaid activities include volunteering, education, and housework

**Fig. 2:**
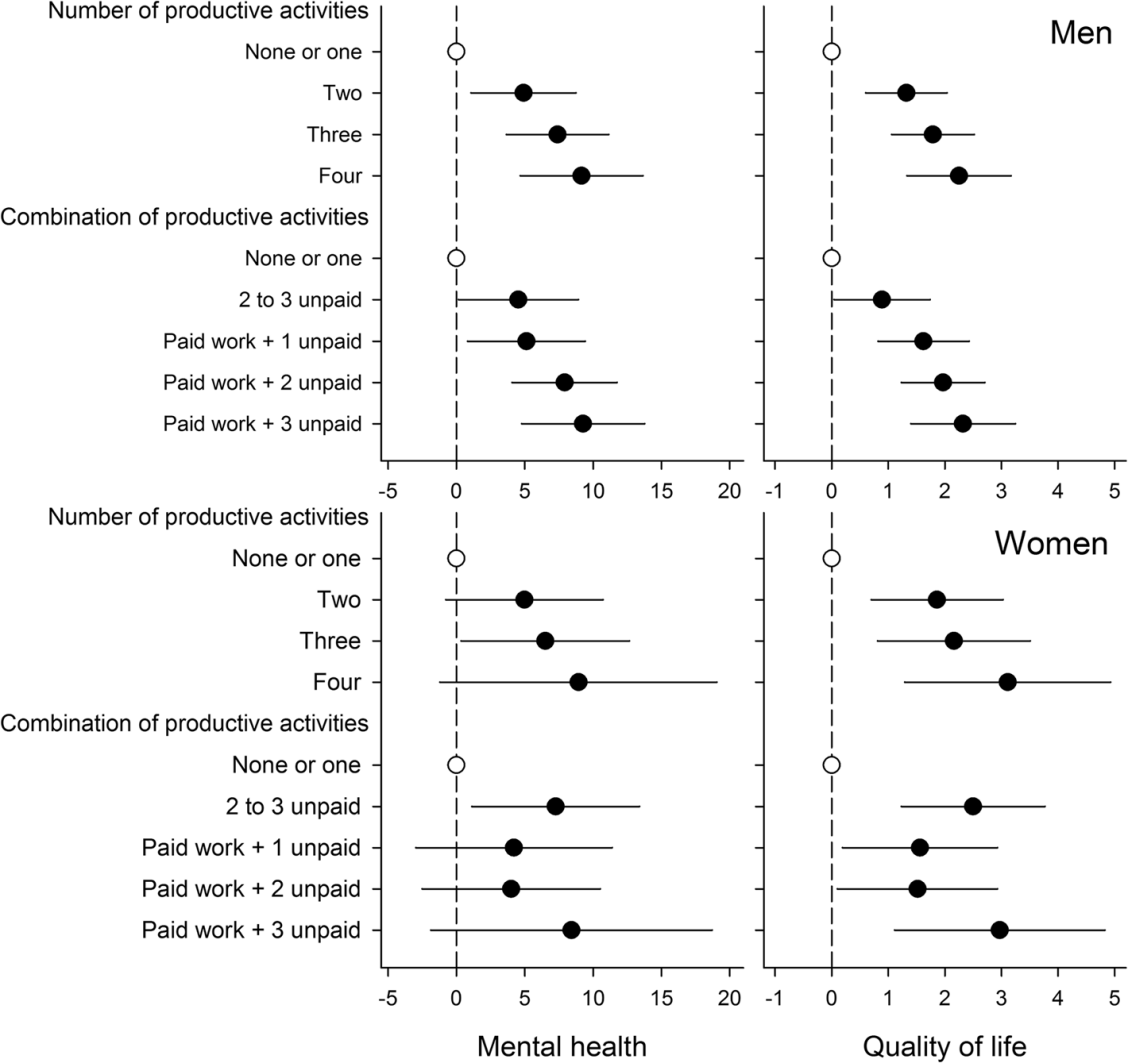
Diversity of productive activities, mental health and quality of life. Adjusted coefficients and 95% confidence intervals from tobit regressions for men (*n* = 818) and women (*n* = 305)

The adjusted results show that the number of productive activities was positively related to mental health in men and QoL in both genders (Fig. 2). Similarly, the analysis of different combinations of productive activities revealed that participants who engaged in more than one productive activity reported better mental health and QoL than those performing no or only one productive activity. In men, paid work in combination with any unpaid activity was linked to better mental health and QoL, with an increase with each additional unpaid activity. In females, scores were highest in women with accumulation of unpaid activities, with the exception of women engaged in all four productive activities who scored highest. Sensitivity analyses for QoL indicate that results remain stable after additional adjustment for mental health (Table S1, Electronic Supplementary Material). Gender did not moderate the association between engagement in a diversity of productive activities and mental health and QoL as none of the tested interactions between gender and diversity of engagement was significant (*p* = 0.42 to 0.95). A tendency for moderation was observed in case of combination of activities and gender for QoL (test for interaction, *p* = 0.07).

## Discussion

This is the first study in the disability setting that explores engagement in productive activities and its link to mental health and QoL and provides clear support for the role enhancement hypothesis. We observed that higher total productivity load and load of paid work were associated with better mental health and QoL in men. Further, moderate engagement in volunteering was positively related to mental health (both genders) and QoL (in women), while household activities and education were not associated with the studied outcomes. Our results support the role enhancement hypothesis as the diversity of productive activities was positively related to mental health (in men) and QoL (both genders). Engagement in paid work in combination with any kind of unpaid activity was linked to enhanced mental health and QoL. The combination of different role engagements and its link to mental health and QoL was inconsistent in women. Our results tentatively suggest a gender-specific impact of paid work on mental health and QoL.

Our results support the assumption that gender roles with respect to productive activities may induce a gender-specific impact of productive activities on mental health and QoL. More specifically, the disability-related exclusion from the labour market may has more serious consequences for males’ than for females’ mental health and QoL. Men who are excluded from paid work may experience feelings of social reward deficiency [13], social exclusion [11], and lack of autonomy [12] that reduce mental health and QoL. Our findings also support the notion that men who are engaged in paid work feel confirmed in their gender role identity as breadwinner [29] and that this confirmation positively affects mental health and QoL. In women, productive activities were mainly linked to QoL and less so to mental health, and paid work was inconsistently associated with the outcomes. Previous studies reported that a majority of employed women perceive paid work as competing with family obligations and source for role conflicts [29]. Although the proportion of women engaged in the Swiss labour market steadily increased over the past 20 years (1996: 70.1%; 2016: 79.5% of women in employable age) [53], there are still considerable gender differences in engagement in unpaid family work and household duties [54]. Competing family and household obligations may reduce the positive effect of paid work on mental health and QoL in employed females in our sample. Age is another potentially relevant personal factor that may modify the associations of productive engagement with mental health and QoL, related to variation in the subjective importance or the societal evaluation of different productive activities over the life course. Future studies may provide insights into the moderating role of age of productive engagement on mental health and QoL.

An important finding is that volunteering may provide additional independent benefits in promoting mental health and QoL in the context of disability. Although many societies have committed to the Convention on the Rights of Persons with Disabilities which foresees equal chances of labour market participation for all individuals [55], persons with physical disabilities face substantial barriers to engage in paid work [24]. To nevertheless achieve a fulfilling live in face of contemporary barriers, evidenced by good mental health and QoL, volunteering may thus provide an important source for basic human needs fulfilment in persons with disabilities, such as appreciation from others or social affiliation [5]. Our results are in line with findings mainly derived from elderly populations [56]. Moreover, the beneficial effect of moderate load of volunteering found in our study was also observed in a longitudinal study including nearly 6000 individuals in the employable age showing that a moderate amount of volunteering (< 100 h/year) predicted a slower decline of QoL in comparison to non-engagement or higher engagement [57]. Notably, engagement in household activities and education were not related to the outcomes under study. As these activities are useful for the individual rather than for a larger group, the beneficial effects resulting from social exchange (e.g., recognition, belonging) might be weaker [23].

In our population with varying degrees of functional capacity, we found large support for the role enhancement hypothesis and approve the notion that the positive outcomes of being productive outweigh potential negative impacts of engagement in a diversity of roles (e.g., role strain, overload or conflict) as claimed by the role strain hypothesis [17]. In contrast to the inconclusive findings from general population samples [14, 18], we observed that being engaged in a diversity of productive activities has beneficial effects on mental health and QoL in persons with physical disabilities, despite the fact that the disabling condition leads to constraints on time, resources and opportunities to engage in productive activities. Our results thus support the assumption that persons who manage to overcome the barriers against engagement profit from the opportunities to experience belonging, skill development, self-efficacy, and recognition from others that ultimately exert beneficial effects on mental health and QoL [6–10].

Our results support the aim of vocational rehabilitation to reintegrate persons with disabilities into paid work, however, strengthen productive activities beyond paid work in persons with physical disabilities may be an equally valuable strategy in persons who face insurmountable barriers to labour market participation. Moreover, it is highly likely that the optimal load of productive activities is individual, depending on a complex interplay between various factors such as the functional capacity, personal characteristics (e.g., self-efficacy, educational background), psychosocial resources (e.g., social network, attributed benefit of paid work), and environmental factors (e.g., social security system, attitudes towards persons with disabilities, access to suitable jobs) [24, 58, 59]. Targeted vocational rehabilitation programs might be an important instrument to increase the individual optimum for engagement in productive activities, for example by strengthening work capacity, personal and psychosocial resources and by reducing environmental barriers. These personal and psychosocial resources may also partly explain the observed associations of engagement in productive activities with mental health and QoL. Future research on the mechanisms behind the identified associations is warranted to develop interventions and policies that strengthen resources important for engagement in productive activities as well as for mental health and QoL in persons with physical disabilities.

### Strengths and limitations

This is the first study to analyse four different types of productive activities and its gender-specific association to mental health and QoL in the disability setting. Major strength of this study are its rigorous epidemiological approach, using a large population-based sample of persons with a physical disability, including established and validated measures to assess mental health and QoL and applying state-of-the-art multivariable statistical methods, taking into account relevant confounders and potential bias due to item- and unit-nonresponse [28]. Importantly, the functional capacity of persons with SCI was adjusted for, thus limiting a bias towards individual capacity. Moreover, we used a theory-based approach and tested clear hypothesis.

Several limitations need to be considered when interpreting the results of this study. Notably, causality between engagement in productive activities and the studied outcomes cannot be inferred and we cannot conclude that increasing the productivity load enhances mental health or QoL in a dose-response relationship. Furthermore, we cannot test whether the occurrence of acute health conditions impacts on current productive engagement and the exclusion of acute health conditions as confounders might lead to bias in observed association. Also, bias due to unmeasured confounders such as acceptance of the disabling condition or personality traits that may be related to the engagement in productive activities as well as to QoL cannot be excluded. Although the analyses are based on a large sample size, the SwiSCI survey is not a census of all persons with SCI in Switzerland and hence, generalizability of the results may be limited. Comparisons of the SwiSCI study sample with traumatic cases in hospital databases provided evidence for a slight underrepresentation of persons with less severe traumatic injuries in the SwiSCI sample [60].

## Conclusion

Our study in the disability setting provides support for the role enhancement hypothesis, as individuals reporting engagement in a diversity of productive activities showed better mental health and QoL. This study supports the aim of vocational rehabilitation to strengthen productive activities beyond paid work in persons with physical disabilities and to consider gender-specific needs and prioritization of productive activities. As the individual optimum of the engagement load depends on functional capacity, general population studies may take functional capacity into account to reduce bias. An in-depth understanding of mechanisms behind the observed associations is still needed to develop interventions and policies that strengthen resources for engagement in productive activities, mental health, and QoL in persons with physical disabilities.

## Abbreviations

FMI: Fraction-missing-information
MHI-5: 5-item Mental Health Inventory
MICE: Multiple imputation by chained eqs.
SCI: Spinal cord injury
SCIM-SR: Spinal Cord Injury Independence Measure for Self-Report
SF-36: 36-item Short Form Health Survey
SwiSCI: Swiss Spinal Cord Injury Cohort Study
USER-P: Utrecht Scale of Evaluation in Rehabilitation-Participation
WHOQoL BREF: World Health Organization Quality of Life Bref

## References

[1] Liu H, Lou VW. Patterns of productive activity engagement as a longitudinal predictor of depressive symptoms among older adults in urban China. Aging Ment Health. 2016:1–8. 10.1080/13607863.2016.1204983 27392120

[2] Kim JH (2013). Productive activity and life satisfaction in Korean elderly women. J Women Aging.

[3] Choi KS, Stewart R, Dewey M (2013). Participation in productive activities and depression among older Europeans: survey of Health, ageing and retirement in Europe (SHARE). Int J Geriatr Psychiatry.

[4] Wahrendorf M, Ribet C, Zins M, Siegrist J (2008). Social productivity and depressive symptoms in early old age-results from the GAZEL study. Aging Ment Health.

[5] Lindenberg S, Frey BS (1993). Alternatives, frames and relative prices: a broader view of rational choice theory. Acta Sociol.

[6] Ryff CD, Singer B (1998). The contour of positive human health. Psychol Inq.

[7] Bowlby J (1969). Attachment and loss.

[8] Bandura A. Social foundations of thought and action Englewood, Cliffs, N.J.: Prentice Hall; 1986.

[9] Mead GH (1934). Mind, self, and society.

[10] Pearlin LI (1989). The sociological study of stress. J Health Soc Behav.

[11] Berkman LF, Glass T, Berkman LF, Kawachi I (2000). Social integration, social networks, social support, and health. Social Epidemiology.

[12] Karasek R, Theorell T (1990). Healthy work.

[13] Siegrist J (2005). Social reciprocity and health: new scientific evidence and policy implications. Psychoneuroendocrin.

[14] Rozario P, Morrow-Howell N, Hinterlong J (2004). Role enhancement and role strain: assessing the impact of multiple productive roles on older caregiver well-being. Res Aging.

[15] Bonfenbrenner U. The ecology of human development. Cambridge, MA: Harvard University Press; 1979.

[16] Sieber SD (1974). Toward a theory of role accumulation. Am Sociol Rev.

[17] Goode WJ (1960). A theory of role strain. Am Sociol Rev.

[18] Baker LA, Cahalin LP, Gerst K, Burr JA (2005). Productive activities and subjective well-being among older adults: the influence of number of activities and time commitment. Soc Ind Res.

[19] Menec VH (2003). The relation between everyday activities and successful aging: a 6-year longitudinal study. J Gerontol B Psychol Sci Soc Sci.

[20] World Health Organization (2011). World report on disability.

[21] Williams R, Murray A (2015). Prevalence of depression after spinal cord injury: a meta-analysis. Arch Phys Med Rehabil.

[22] Dijkers MP (2005). Quality of life of individuals with spinal cord injury: a review of conceptualization, measurement, and research findings. J Rehabil Res Devel.

[23] Siegrist J, Fekete C (2016). Fair opportunities, social productivity and wellbeing in disability: towards a theoretical foundation. J Rehabil Med.

[24] Reinhardt JD, Post MW, Fekete C, Trezzini B, Brinkhof MW (2016). Labor market integration of people with disabilities: results from the Swiss spinal cord injury cohort study. PLoS One.

[25] Reinhardt JD, Ballert C, Brinkhof MW, Post MW (2016). Perceived impact of environmental barriers on participation among people living with spinal cord injury in Switzerland. J Rehab Med..

[26] Young AE, Murphy GC (2009). Employment status after spinal cord injury (1992-2005): a review with implications for interpretation, evaluation, further research, and clinical practice. Int J Rehab Res.

[27] Lidal IB, Huynh TK, Biering-Sorensen F (2007). Return to work following spinal cord injury: a review. Disabil Rehabil.

[28] Bickenbach J, Officer A, Shakespeare T, von Groote P (2013). International perspectives on spinal cord injury.

[29] Simon RW (1995). Gender, multiple roles, role meaning, and mental health. J Health Soc Behav.

[30] Molarius A, Granstrom F, Linden-Bostrom M, Elo S (2014). Domestic work and self-rated health among women and men aged 25-64 years: results from a population-based survey in Sweden. Scand J Public Health.

[31] Plaisier I, de Bruijn JG, Smit JH, de Graaf R, Ten Have M, Beekman AT, van Dyck R, Penninx BW (2008). Work and family roles and the association with depressive and anxiety disorders: differences between men and women. J Affect Disord.

[32] The World Bank: Labor force participation rate, female (% of female population ages 15+) (modeled ILO estimate). 2017. https://data.worldbank.org/indicator/SL.TLF.CACT.FE.ZS Accessed 20 June 2018.

[33] Post MW, Brinkhof MW, von Elm E, Boldt C, Brach M, Fekete C, Eriks-Hoogland I, Curt A, Stucki G (2011). Design of the Swiss Spinal Cord Injury Cohort Study. Am J Phys Med Rehabil.

[34] Brinkhof MW, Fekete C, Chamberlain JD, Post MW, Gemperli A (2016). Swiss national community survey on functioning after spinal cord injury: protocol, characteristics of participants and determinants of non-response. J Rehabil Med.

[35] Fekete C, Segerer W, Gemperli A, Brinkhof MW (2015). Participation rates, response bias and response behaviours in the community survey of the Swiss spinal cord injury cohort study (SwiSCI). BMC Med Res Methodol.

[36] Post MW, van der Zee CH, Hennink J, Schafrat CG, Visser-Meily JM, van Berlekom SB (2012). Validity of the Utrecht scale for evaluation of rehabilitation-participation. Disabil Rehabil.

[37] World Health Organization (1992). ICD-10 classifications of mental and behavioural disorder: clinical descriptions and diagnostic guidelines.

[38] Diener E, Suh EM, Lucas RE, Smith HL (1999). Subjective well-being: three decades of progress. Psychol Bull.

[39] Ware JE, Sherbourne CD (1992). The MOS 36-item short-form health survey (SF-36). I. Conceptual framework and item selection. Med Care.

[40] van Leeuwen CM, van der Woude LH, Post MW (2012). Validity of the mental health subscale of the SF-36 in persons with spinal cord injury. Spinal Cord.

[41] Ware JE, Snow KK, Kosinski M, Gandek B (1993). SF-36 Health survey. Manual and interpretation guide.

[42] World Health Organization (2004). The World Health Organization quality of life (WHOQOL)-BREF.

[43] Geyh S, Fellinghauer BA, Kirchberger I, Post MW (2010). Cross-cultural validity of four quality of life scales in persons with spinal cord injury. Health Qual Life Outcomes.

[44] Migliorini C, Tonge B, Taleporos G (2008). Spinal cord injury and mental health. Aust N Z J Psychiatry.

[45] Noreau L, Dion SA, Vachon J, Gervais M, Laramee MT (1999). Productivity outcomes of individuals with spinal cord injury. Spinal Cord.

[46] Prodinger B, Ballert CS, Brinkhof MW, Tennant A, Post MW (2016). Metric properties of the spinal cord Independence measure - self report in a community survey. J Rehab Med.

[47] Fekete C, Eriks-Hoogland I, Baumberger M, Catz A, Itzkovich M, Luthi H, Post MW, von Elm E, Wyss A, Brinkhof MW (2013). Development and validation of a self-report version of the spinal cord Independence measure (SCIM III). Spinal Cord.

[48] White IR, Royston P, Wood AM (2011). Multiple imputation using chained equations: issues and guidance for practice. Stat Med.

[49] Carpenter JR, Kenward MG. Multiple imputation and its application. Hoboken: Wiley; 2013.

[50] Cuzick J (1985). A Wilcoxon-type test for trend. Stat Med.

[51] Tobin J (1958). Estimation of relationships for limited dependent variables. Econometria.

[52] Li KH, Raghunatan TE, Rubin DB (1991). Large-sample significance levels from multiply imputed data using moment-based statistics and an F reference distribution. J Am Stat Assoc.

[53] Office of Federal Statistics (2017). Schweizerische Arbeitskräfteerhebung (SAKE). [Swiss Labour Force Survey].

[54] Office of Federal Statistics (2017). Familien in der Schweiz: Statistischer Bericht. [Families in Switzerland: Statistical report].

[55] United Nations (2006). Convention of the rights of persons with disabilities.

[56] Jenkinson CE, Dickens AP, Jones K, Thompson-Coon J, Taylor RS, Rogers M, Bambra CL, Lang I, Richards SH (2013). Is volunteering a public health intervention? A systematic review and meta-analysis of the health and survival of volunteers. BMC Public Health.

[57] Hao Y (2008). Productive activities and psychological well-being among older adults. J Gerontol B Psychol Sci Soc Sci..

[58] Fadyl JK, McPherson KM (2010). Understanding decisions about work after spinal cord injury. J Occup Rehabil.

[59] Anderson D, Dumont S, Azzaria L, Le Bourdais M, Noreau L. Determinants of return to work following spinal cord injury: a literature review. J Voc Rehabil 2007;27:57–68.

[60] Chamberlain JD, Ronca E, Brinkhof MW (2017). Estimating the incidence of traumatic spinal cord injuries in Switzerland: using administrative data to identify potential coverage error in a cohort study. Swiss Med Wkly.

